# Mixed effectiveness of rTMS and retraining in the treatment of focal hand dystonia

**DOI:** 10.3389/fnhum.2015.00385

**Published:** 2015-07-09

**Authors:** Teresa J. Kimberley, Rebekah L. S. Schmidt, Mo Chen, Dennis D. Dykstra, Cathrin M. Buetefisch

**Affiliations:** ^1^Program in Physical Therapy, Brain Plasticity Laboratory, Department of Physical Medicine and Rehabilitation, University of MinnesotaMinneapolis, MN, USA; ^2^Department of Physical Medicine and Rehabilitation, University of MinnesotaMinneapolis, MN, USA; ^3^Department of Neurology and Rehabilitation Medicine, Emory UniversityAtlanta, GA, USA

**Keywords:** transcranial magnetic stimulation, writer's cramp, rehabilitation, sensory, human, neuromodulation

## Abstract

Though the pathophysiology of dystonia remains uncertain, two primary factors implicated in the development of dystonic symptoms are excessive cortical excitability and impaired sensorimotor processing. The aim of this study was to determine the functional efficacy of an intervention combining repetitive transcranial magnetic stimulation (rTMS) and sensorimotor retraining. A randomized, single-subject, multiple baseline design with crossover was used to examine participants with focal hand dystonia (FHD) (*n* = 9). Intervention: 5 days rTMS + sensorimotor retraining (SMR) vs. Five days rTMS + control therapy (CTL) (which included stretching and massage). The rTMS was applied to the premotor cortex at 1 Hz at 80% resting motor threshold for 1200 pulses. For sensorimotor retraining, a subset of the Learning-based Sensorimotor Training program was followed. Each session in both groups consisted of rTMS followed immediately by 30 min of the therapy intervention (SMR or CTL). Contrary to our hypothesis, group analyses revealed no additional benefit from the SMR training vs. CTL. When analyzed across group however, there was significant improvement from the first baseline assessment in several measures, including tests of sensory ability and self-rated changes. The patient rated improvements were accompanied by a moderate effect size suggesting clinical meaningfulness. These results provide encouragement for further investigation of rTMS in FHD with a need to optimize a secondary intervention and determine likely responders vs. non-responders.

## Introduction

Focal dystonia is a movement disorder that can affect any body part and severely impair a person's ability to function in their daily life. In focal hand dystonia (FHD) there is an involuntary activation of agonist and antagonist muscles in the hand and forearm. In task specific hand dystonia, the dystonia manifests when a person attempts to execute specific tasks such as writing, typing, or playing a musical instrument. Most often these tasks are related to a repetitive action, often done in the context of an occupation, thus, impairing the ability to work and reducing quality of life. The individual with FHD otherwise has a normal neurological exam and normal function of the hand with other tasks. Although the pathophysiology of FHD remains unclear, and may differ among the different types of dystonias, there is considerable evidence for abnormal inhibition in primary motor cortex (M1), premotor cortex (PMC), spinal cord, and brainstem (Siebner et al., 1999; Butefisch et al., 2005; Hallett, 2006, 2011; Quartarone et al., 2014). For example, it has been shown that participants with task specific dystonia have disturbances in task dependent inhibition in M1 (Butefisch et al., 2005) and intracortical inhibition (Kimberley et al., 2009). While dystonia is mainly a motor problem, mild abnormalities of sensation have been reported in patients with dystonia, even in body parts not affected by the dystonia. There is also evidence to suggest that the problem may be related to faulty sensorimotor integration (for review, Abbruzzese and Berardelli, 2003; Quartarone et al., 2014). Another abnormality in FHD is that of plasticity. It has been demonstrated that decreases in M1 inhibition facilitate the induction of plasticity (Hess et al., 1996; Di Lazzaro et al., 2006), thus, the abnormally increased plasticity in dystonia could be related to the abnormally decreased motor cortical inhibition that is found in patients with dystonia (Hallett, 2011).

The evidence for abnormal inhibition in patients with dystonia is derived from experiments using transcranial magnetic stimulation (TMS) to M1 as a testing tool to measure the level of intracortical inhibition in the GABAnergic system (Ridding et al., 1995; Butefisch et al., 2005). Most related to this proposal, the cortical silent period (CSP) is attributed to both spinal and cortical inhibition and is a test of GABA_B_ activity (Chen et al., 1999; Kimberley et al., 2009). CSP is the duration of EMG quiescence following a TMS pulse to M1 that is superimposed upon a low level of voluntary muscle contract in the muscle of interest (Kimberley et al., 2009).

While TMS provides a means to measure these abnormalities in M1 excitability, when applied repetitively (repetitive TMS, rTMS) it can also be used as a tool to modulate excitability of the stimulated neuronal tissue. When applied at low frequencies, it results in increased inhibition in the stimulated area of the brain that persists for more than 20 min post intervention (Chen et al., 2003). This rTMS protocol has been applied in persons with FHD in a single session and a temporary reduction in dystonic posturing and increased inhibition was recorded with a maximal effect when stimulation was applied to the PMC (Murase et al., 2005). These findings have been extended by applying low-frequency (1 Hz) rTMS to the PMC in persons with FHD for 5 days, resulting in improved handwriting velocity and changes in brain excitability (Borich et al., 2009). Additional work examined whether rTMS applied during performance of an active, but non-dystonic, hand “writing” task would enhance the beneficial effects (Kimberley et al., 2013). The multiple sessions of rTMS strengthened intracortical inhibition causing a prolongation of CSP after 3 days of intervention and pen force was reduced at day 1 and 5; notably, 68% of patients self-reported as “responders” after 5 days of intervention, suggesting potential beneficial effect of the inhibitory neuromodulation (Kimberley et al., 2013). Together, evidence suggests that rTMS induced PMC inhibition is an encouraging approach for neuromodulation therapy in people with FHD. Neuromodulation alone may not be adequate to produce clinically meaningful changes, however.

Beyond botulinum toxin, which has a variable and temporary benefit, there are few opportunities for treatment for people with FHD. Rehabilitation interventions based upon the hypothesis of aberrant learning and maladaptive plasticity have had some modest success [for review (Altenmuller and Jabusch, 2010)]. Sensorimotor training is one of these interventions that has been shown to modify brain activation and produce some functional improvements (Byl and McKenzie, 2000; Zeuner et al., 2002, 2003; Byl et al., 2003, 2009). The objective of the training is to redefine spatial and temporal processing capacities through guided activities that emphasize different aspects of sensory feedback (e.g., somatosensation, proprioception, kinesthesia) in order to improve sensorimotor integration and restore task-specific skills. A significant limitation of this training is the duration of treatment, however, which ranges from 8 to 12 weeks and requires several hours of therapy/day. Additionally, although improvements have been shown to occur in some cases, participants do not achieve premorbid levels of function and it is not universally beneficial.

The purpose of this study was to investigate whether the pairing PMC rTMS with sensorimotor retraining is more effective in improving dystonia and abnormal inhibition when compared to PMC rTMS with a control intervention. Combining neuromodulation treatment by means of rTMS or transcranial direct current stimulation with motor training is an approach that has been used in the rehabilitation of motor stroke (Hao et al., 2013) and builds on the evidence that plastic changes are either enhanced or blocked depending on the rTMS protocol (Butefisch et al., 2004). Considering the evidence for abnormally enhanced plasticity in people with FHD, executing a sensorimotor training in a more normal neurophysiological state (i.e., improved M1 inhibition) may enhance the effect of the training. Given the previous work that demonstrated increased inhibition in people with FHD following rTMS to PMC (Borich et al., 2009), as well as positive clinical changes from a prolonged intervention protocol, we hypothesized that rTMS applied to the PMC followed by sensorimotor training (SMR) would result in reduced dystonia symptoms and increased M1 inhibition, compared to rTMS alone.

## Methods

### Experimental design

A randomized, crossover study design was used. Participants were randomly assigned to one of two initial treatment phases: rTMS + sensorimotor retraining (SMR) or rTMS with no specialized retraining. In order to control for the unspecific effects of an intervention such as emotional support, attention etc., subjects in the rTMS with no specialized retraining condition were exposed to a control therapy (CTL, rTMS + CTL) with non-specific stretching and massage. Each phase schedule consisted of 2 days of baseline testing, five intervention sessions, 2 days of post-test testing and one follow-up session (1-week after post-test) (Table 1). After the completion of the first phase of intervention, follow-up test, and a 1-month washout period, participants crossed over to the other intervention. In other words, the people that were randomly assigned to SMR first crosses over to receive CTL (SMR-CTL), and the people that received the CTL first, crosses to receive SMR (CTL-SMR).

**Table 1:** **Study schedule example**.

	**Day 1**	**Day 2**	**Day 3**	**Day 4**	**Day 5**
Week 1	Baseline 1	Baseline 2	Intervention	Intervention	Intervention
Week 2	Intervention	Intervention	Post-test 1	Post-test 2	
Week 3				Follow-up	

Participants completed each phase of treatment according to a schedule that included two baseline sessions, five intervention sessions, two post-test sessions and a follow-up session 1-week later.

The baseline and post-test testing sessions consisted of a series of behavioral and motor cortex corticospinal excitability outcome measures. Behavioral outcome measures included digitized and physician rated handwriting, self-reported symptom severity, clinical hand dystonia assessment, and sensory testing. The TMS measure used to investigate intracortcial inhibition was the CSP as this has been found to be the most sensitive to change (Daskalakis et al., 2006). Digitized handwriting and TMS measures were collected at each testing session (two baseline and two post-test) while all other measures were collected once during baseline testing and once during post-test testing. The crossover design was selected to maximize participant recruitment in this rare disorder and allow all participants to receive both therapies. The two baseline- and post-test-testing sessions were chosen to determine a more stable response to the TMS and handwriting measures by averaging over 2 days.

### Participants

**Figure 1:**
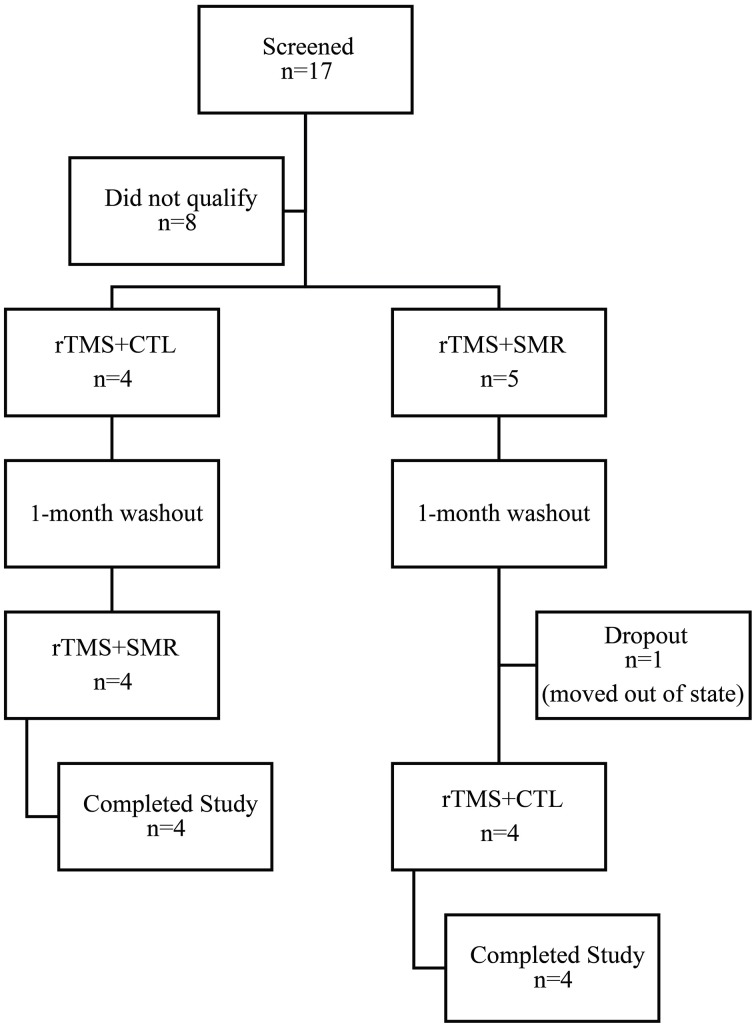
**Consort diagram of screened and enrolled participants**. Participants were randomly assigned to either the rTMS + SMR or rTMS + CTL intervention first and then crossed over to receive the other intervention after 1-month washout. (SMR, sensorimotor retraining intervention; CTL, control intervention; rTMS, repetitive transcranial magnetic stimulation).

Nine participants (3 females; mean age: 46 ± 10.6 years) (Table 2) with FHD were recruited and eight completed the entire study. One subject dropped out after the first phase of intervention due to moving out of state (Figure 1). Exclusion criteria included any neurologic conditions other than dystonia, botulinum injection within the past 4 months, medications with effects on the central nervous system and contraindications to rTMS (Rossini et al., 2007). Participants gave written, informed consent prior to participation according to the Declaration of Helsinki (World Medical Association, 2013) and study approval was granted by the University of Minnesota Clinical and Translational Science Institute and Institutional Review Board.

**Table 2:** **Participant demographics**.

**Participant**	**Group**	**Age**	**Duration of Sx (Years)**	**Gender**	**Side of FHD**	**Handedness**	**Medication use**	**Clinical pattern**
1	CTL-SMR	46	9	M	R	R	N	First and second digit
2	SMR-CTL	60	9	M	R	R	Y	First digit extension and adduction
3	SMR-CTL	50	3	M	L	L	Y	grip, wrist extensor spasms during writing/typing
4	CTL-SMR	53	25	M	R	R	Y	Muscle weakness, writing/typing affected
5	SMR-CTL	51	4	M	R	R	Y	First and second digit flexion, radial forearm and triceps weakness
6	SMR-CTL	55	15	F	R	R	Y	First digit adduction, second digit flexion
7	CTL-SMR	40	5	F	L	L	N	Second and third digit flexion and extension
8	CTL-SMR	38	3	M	R	L	N	Fifth digit MCP and DIP extension
9	SMR-CTL	25	8	F	L	L	Y	Fourth digit, overall pain

M, male; F, female; R, right; L, left; N, no; Y, yes; y, years; Sx, symptoms; FHD, focal hand dystonia; MCP, Metacarpophalangeal; DIP, Distal Interphalangeal; CTL, control; SMR, sensorimotor retraining.

### Behavioral measures

#### Participant rated measures

Symptom severity was assessed using the global rating of change (GROC) (Kamper et al., 2009) and Arm Dystonia Disability scale (ADDS) (Fahn, 1989) and SF-36 (Ware et al., 2000). For the GROC, participants were asked to identify between one and three functions most impacted by FHD. At post-test 1 and follow-up, they were then asked to select a rating of perceived change that represented the level of function compared to baseline. Perceived change consisted of a ±7 point Likert scale (+7 = a very great deal better, 0 = no change, −7 = a very great deal worse) (Siebner et al., 1999; Borich et al., 2009). The ADDS was adapted from previous work (Fahn, 1989) where participants completed a survey of task difficulty for activities such as writing, handling utensils, and buttoning on a scale of 1–4 (1 = no difficulty, 4 = not able or marked difficulty). Participants completed the full SF-36 assessment with subsections of interest: “physical functioning” and “role of limitations due to physical health” and “emotional well-being.” ADDS and SF-36 were collected at baseline 1, post-test 1, and follow-up.

#### Clinical hand dystonia assessment

Video recordings were made as participants wrote on a pad of paper with pen. Participants were asked to draw a series of 10 loops across the pad of paper followed by *“The dog is barking”* and their signature, each repeated four times. A physician blinded to participant allocation rated recordings. Scoring criteria were adapted from a standardized writer's cramp rating scale (WCRS) (Wissel et al., 1996), rating pathological flexion or extension at the wrist, fingers and elbow, presence of tremor, dystonic posture, writing speed and latency of dystonic symptoms. Final scores are expressed as two separate ratings listed as a movement score and speed score.

#### Handwriting

Digitized handwriting was assessed at each baseline, post-test and follow up visit. Handwriting samples were collected using a computerized tablet (WACOM Co., Ltd., japan) with MovAlyzeR® (Neuroscript LLC, Tempe, AZ) hardware and software. Participants used a custom modified digitized pen (Kiko Software, Netherlands) to write in a self-selected pace and style on the tablet with real-time visual feedback. Writing tasks included *“My country tis of thee”* at a self-selected pace, repeated eight times. Data were sampled at 215 Hz (resolution: 5080 lpi, accuracy: ±0.01 pressure range: 0–800 g). Writing samples were segmented by points of minimal velocity into single strokes for analysis. Variables of interest for each stroke were automatically calculated within the software and included: mean peak vertical velocity (Zeuner et al., 2007), axial pen pressure, and normalized jerk which is a measure of dysfluency (Caligiuri et al., 2006, 2009). Data for each measure was exported for statistical analysis. One participant that did not display symptoms affecting handwriting and did not participate in the handwriting analysis.

#### Sensory testing

Sensory discrimination was assessed at baseline, post-test and follow-up. Examinations included two point discrimination and the Byl–Cheney–Boczai Sensory Discriminator (BCB) (Byl et al., 2002). Two-point discrimination threshold was completed using a Disk-Criminator™. Participants were asked to reply “one” or “two” after each presentation. Static and dynamic stimuli were presented to the index and ring fingers bilaterally, meaning it was presented as a static stimuli or it was slowly swept across the skin (dynamic) (Dellon and Kallman, 1983). The BCB test was completed using a custom-made set of cubes with metal beads arranged in unique shapes such as a circle, triangle, and arrow. Each cube was fitted in a wood platform and the participant's finger passed across the cube by the examiner. Participants were then given a visual list of shapes and asked to select the corresponding shape they felt. The index and ring finger were tested bilaterally. The BCB has been designed to assess somatosensory function and sensory discrimination in a clinical setting (Byl et al., 2002).

### TMS

Corticospinal excitability was assessed with the CSP. The CSP was selected as the TMS outcome measure of interest because it is known to be altered in people with FHD (Kimberley et al., 2009) and has been shown to be the most reliable measure of excitability (Daskalakis et al., 2006). For TMS testing and intervention, participants were comfortably seated in a semi-reclined chair. The hand region has previously been described along a mid-hypotenuse line from marks 5 cm lateral and 5 cm anterior to the vertex with a 45° posterolateral orientated TMS coil (Borich et al., 2009). This measurement was used to guide coil placement over the hand motor region. TMS evoked electromyography (EMG) data were collected from the first dorsal interosseous (FDI) muscle on the affected hand. Silver chloride disk electrodes were placed on the target muscle in a belly-tendon montage with a bandpass filter 20–20,000 Hz (Cadwell Laboratory, Washington). To find the optimal position of the TMS coil (hotspot) to activate the FDI muscle, a 70-mm figure-of-eight TMS coil connected to a Magstim BiStim^2^ magnetic stimulator (Magstim Co., Whitland, UK) was used. Single-pulse TMS pulses were delivered while the participant was at rest until a coil position was identified that elicited the largest motor evoked potentials (MEP) with the least intensity of the maximum stimulator output. The intensity was then decreased until the minimum intensity to elicit an MEP amplitude greater than 50 μV (peak-to-peak) in at least three of five trials in the resting target muscle was determined and defined as the resting motor threshold (RMT) (Rossini et al., 1999).

CSP testing was completed during a voluntary isometric contraction of the target muscle whereby the MEP is followed by a short duration of EMG quiescence. The maximal voluntary contraction for finger abduction was recorded using a custom strain gauge placed around the index finger. Real-time visual feedback was given on a laptop screen to project the force produced by the participant and 20% of the maximum of three trials was calculated and displayed on a target line. For the CSP, participants were asked to contract until the target line was met, then a single TMS pulse was delivered to the hotspot at 120% RMT. Ten trials were collected with a short rest period to prevent fatigue.

CSP duration was calculated in milliseconds (ms). CSP EMG data were first rectified, and then a 10-ms moving average calculation was applied to the data. The onset of the CSP was set as the time point of the delivery of the TMS stimulus. The average of the pre-stimulus moving average data (−25 to 0 ms) was used as a threshold to determine the off-set of the CSP, defined as the point that the moving average value returned to the pre-stimulus level. The average of the 10 trials CSP was calculated.

All interventions sessions included rTMS, which consisted of 1200 pulses (20 min) delivered by a Magstim Rapid^2^ magnetic stimulator (Magstim Co., Whitland, UK) at 1 Hz with the intensity of 80% of RMT to PMC which replicated previous studies assess the effect of rTMS without rehabilitation intervention (Borich et al., 2009; Kimberley et al., 2013, 2015). The PMC was defined as 1 cm medial and 2 cm rostral to the hotspot of the first dorsal interosseous muscle (Fink et al., 1997).

### Training

Sensorimotor retraining was based on aspects of the Learning-based Sensorimotor Training program (Byl et al., 2003). The program was administered for 30 min immediately following the rTMS during the training phase. Participants were guided though supervised practice of sensory discrimination training. For example, with vision obstructed, the participant was required to discriminate grades of sandpaper as well as perform various stereognosis and graphesthesia tasks. Sensory decimation training focused primarily on the digits associated with the individual's clinical pattern (Table 2) but was also performed with all digits, palm and dorsum and on the unimpaired hand. Tasks were primarily dynamic, meaning, the participant actively moved their digit across a surface and then used another digit to move across different choices to select the matching texture. Other tasks required the participant to select and pull out small safety pins from bowls of rice. Tasks were made progressively more difficult throughout the treatment.

The CTL intervention was also delivered immediately following the rTMS and consisted of 30 min of active and passive generalized stretching to wrist, shoulder and finger muscles and massage to the wrist, hand and shoulder musculature. All participants were instructed to avoid tasks most affected by dystonic symptoms as much as possible for the duration of the treatment. No home exercise instruction was given.

### Data processing

For measures that were collected on two pre-test sessions or two post-test sessions (handwriting and TMS), averages were calculated from the two baselines or the two post-tests, respectively. This approach was intended to represent the true baseline and post-test values. To make the data comparable between groups for all data points, the normalized change scores were calculated using a linear transformation as: Normalized change score = (*X*_*post*_ − *X*_*baseline*_)/*X*_*baseline*_ where, *X*_*post*_ is the average value of the two post-tests; *X*_*baseline*_ is the mean value of the two baselines. The only exception to the change score analysis was the WCRS, which was assessed with raw scores, due to the potential of the baseline to have a 0 score.

### Statistical analysis

#### Group analysis

Pre-planned comparisons for all dependent measures (Table 3 for list) included two-factor repeated measures analysis of variance (RM ANOVA) to determine a group (SMR and CTL) × time [change from baseline (average of both baseline days) to post-test (average of post-test days 1 and 2) and follow-up] interaction of change from baseline for each treatment phase (*p* < 0.05). If a lack of group effect was found, data would be collapsed between groups to assess change from baseline to both follow up 1 and follow up 2 to determine longitudinal effect. Normality was assessed with Shapiro–Wilk *W*-test (*p* < 0.05). Initial comparison was with RM ANOVA (*p* < 0.05) and then, given lack of normality when data were collapsed, Friedman analysis of variance by ranks (*p* < 0.05) was applied to determine longitudinal effect over time, including both phases of the experiment. In this case, change from initial baseline to two assessment points (1. follow-up of phase one, 2. follow-up of phase two) was calculated and used for the Friedman test. Additionally, effect sizes are expressed through Kendall's coefficient of concordance which is a non-parametric statistic associated with the Friedman test that makes no assumptions regarding the probability distribution (Kendall and Smith, 1939). As indicated, *post hoc* Wilcoxon signed rank test corrected for multiple comparisons (two repeated measures, *p* < 0.025) was used to accompany Friedman analysis.

**Table 3:** **Results repeated measures ANOVA**.

**Outcome**	***F* (*n*, *df*)**	**Group × time**	**Sphericity**
CSP	1.132 (8, 2)	0.336	Assumed
2pt Non-dom ring	2.743 (8, 2)	0.081	Assumed
2pt Non-dom index	0.248 (8, 2)	0.782	Assumed
2pt Dom index	0.241 (8, 2)	0.787	Assumed
2pt Dom ring	1.028 (8, 2)	0.370	Assumed
ADDS	0.272 (8, 2)	0.763	Assumed
2pt Static dom index	0.651 (8, 1.4)	0.476	Rejected, GG *p* = 0.014
2pt Static dom ring	0.548 (8, 1.4)	0.524	Rejected, GG *p* = 0.018
2pt Static non-dom index	0.008 (8, 1.3)	0.964	Rejected, GG *p* = 0.004
2pt Static non-dom ring	0.524 (8, 2)	0.598	Assumed
BCB right ring	1.278 (8, 2)	0.294	Assumed
Emotional well-being	0.338 (8, 1.4)	0.635	Rejected, GG *p* = 0.012
Physical functioning	0.115 (8, 1.2)	0.785	Rejected, GG *p* = 0.0001
Role of limitations due to physical health	1.988 (8, 1.1)	0.177	Rejected, GG *p* = 0.0001
Handwriting jerk	0.373 (8, 2)	0.693	Assumed
Handwriting pressure	0.553 (8, 1.4)	0.523	Rejected, GG *p* = 0.31
Handwriting velocity	0.161 (8, 1.0)	0.705	Rejected, GG *p* = 0.0001
WCRS movement score	0.337 (8, 2)	0.717	Assumed
WCRS writing speed score	2.302 (8, 1.5)	0.135	Rejected, GG *p* = 0.045

*CSP, cortical silent period; Dom, dominant; Non-dom, non-dominant; 2pt, two-point discrimination; ADDS, Arm dystonia disability scale; BCB, Byl–Cheney–Boczai Sensory Discriminator; WCRS, writer's cramp rating scale; GG, Greenhouse–Geisser correction for sphericity. No significant interactions detected*.

The use of effect size in clinical intervention studies has been increasingly advocated for to improve interpretation of results, given the large variability and small sample sizes that often accompany interventional based studies (Ottenbacher and Maas, 1999; Musselman, 2007; Gomes-Osman and Field-Fote, 2015).

#### Single subject analysis

Given the small sample size and the variable response to rTMS (López-Alonso et al., 2014), *a priori* defined single subject analyses were also conducted. Single subject responses were evaluated using a 2 SD band method (Backman et al., 1997; Deng et al., 2013). GROC scores were assessed using minimal clinically important differences (MCID) with changes of ±5 considered clinically meaningful (Kamper et al., 2009). Physician's ratings of WCRS >1 point was considered meaningful. Two-point discrimination changes were considered meaningful if ≥2 testing levels on the Disk-Criminator™.

## Results

### Group results

#### Effect of the two different interventions on behavior and corticospinal excitability

Results of the two factor RM ANOVA revealed no significant intervention (CTL vs. SMR) × time (Pre-test, Post-test, Follow up) interactions in any measure, indicating there was no superior benefit or neurophysiologic change to the sensorimotor retraining intervention with rTMS compared to control with rTMS (Table 3).

**Figure 2:**
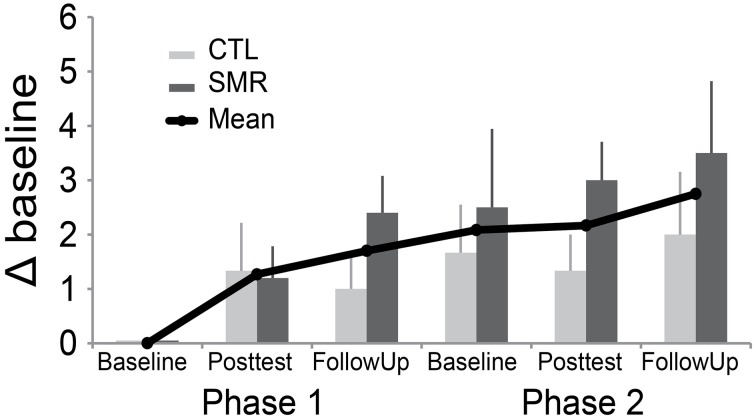
**Global rating of change (GROC) scores from first baseline to all subsequent assessments**. This graph demonstrates mean change (±SE) over time, excluding one participant (#7) who appeared to misunderstand the rating (see text). Note, all individuals received both interventions. CTL-SMR (light gray) received the rTMS + CTL first and then the rTMS + SMR in Phase 2. The SMR-CTL (dark gray) group received rTMS + SMR first and then rTMS + CTL in Phase 2. GROC was assessed at one baseline, post-test and follow up for each phase. (SMR, sensorimotor retraining; rTMS, repetitive transcranial magnetic stimulation; CTL, control).

In contrast to our expectations, mean GROC scores did not return to zero following the washout period, which would indicate a return to the previous level of function (Figure 2). This suggests a carryover effect from the participants' first intervention into the beginning of the second intervention. There was no difference in carryover effect between treatments.

One participant (#7) in the CTL-SMR group reported a worsening of symptoms at the first post-test (after CTL) that returned to normal at follow-up (GROC change of -4). This participant reported significant worsening of symptoms (GROC change of −4) in the subsequent SMR phase baseline and a further worsening at both post-test and follow-up (GROC change of −6). However, it appeared that the participant did not understand the rating instructions with the GROC measure, and there were conflicting responses when compared to the other self-reported measures. For example, the subject did not report worsening in the ADDS assessment which asked the same question regarding function as the identified GROC variable. For qualitative assessment this participant (#7) was removed from the visual inspection of the GROC in Figure 2, due to concerns mentioned, but is included for all other analyses.

**Figure 3:**
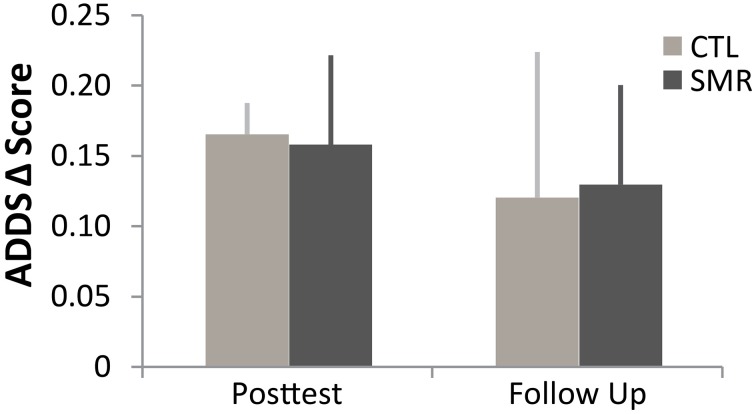
**Arm Dystonia Disability Scale (ADDS) in first phase of treatment (CTL** ***n*** **= 4 in dark gray, SMR** ***n*** **= 5 in light gray)**. Regardless of intervention, all subjects reported improvement in self-perceived function. Mean (±SE). ADDS was tested at one baseline, post-test and follow up for each phase. (SMR, sensorimotor retraining, rTMS, repetitive transcranial magnetic stimulation, CTL, control).

Overall, the results of ADDS (Figure 3) suggest a perceived improvement by participants during the first treatment, regardless of group (all participants).

#### Overall effect of interventions on corticospinal excitability and behavior

Given lack of overall group effect, data were collapsed across groups to determine the effects of the treatment across the entire experiment within each participant. For this analysis, normalized change scores were calculated from Phase 1 (average of the two baseline assessments for handwriting and TMS measures) to Follow up 1 and Follow up 2. Data were found to be non-normal (*p* < 0.05 Shapiro–Wilk *W*-test). Friedman test was conducted to evaluate differences in medians of the three test points. The results are listed in Table 4. Of note, there were significant findings in two self-reported measures; emotional well-being *X*^2^(2, *N* = 8) = 11.21, *p* = 0.004, Kendall's coefficient of concordance = 0.70; and ADDS: *X*^2^(2, *N* = 8) = 6.1, *p* = 0.048, Kendall's coefficient of concordance = 0.38, as well as four different sensory tests (see Table 4). Size of effect were considered: 0.1 = small, 0.3 = medium, and >0.5 indicate a large effect (Field, 2009) (Table 4), thus the effects were large and medium, respectively. *Post hoc* Wilcoxon signed rank test corrected for multiple comparisons (two repeated measures, *p* < 0.025) revealed improvements in 2pt discrimination of the dominant ring finger (*N* = 8, *Z* = 2.39, *p* < 0.017) between baseline and follow up 1 and SF36 emotional well-being between baseline and Follow up 2 (*N* = 8, *Z* = 2.37, *p* < 0.018), and BCB ring finger (*N* = 8, *Z* = 2.38, *p* < 0.017), with a trend in ADDS (*p* = 0.028).

**Table 4:** **Results collapsed across group assessing change over time**.

**Measure**	**Friedman's *X*^2^ (2, *N* = 8)**			**Baseline to follow-up 1**		**Baseline to follow-up 2**	
	**Chi-square**	***p*-values**	**Kendall's W**	**Mean (95% CI)**	***p*-values**	**Mean (95% CI)**	***p*-values**
2pt Dynamic dom index	7.182	0.028^*^	0.449	−0.31 (−0.62, 0.00)	0.050	−0.32 (−0.66, 0.01)	0.074
2pt Dynamic dom ring	2	0.368	0.125	−0.46 (−0.33, 0.24)	0.713	−0.17 (−0.47, 0.13)	0.168
2pt Dynamic non-dom index	1.727	0.422	0.108	−0.18 (−0.60, 0.25)	0.309	−0.13 (−0.61, 0.35)	0.462
2pt Dynamic non-dom ring	3.769	0.152	0.236	−0.26 (−0.50, −0.02)	0.062	−0.36 (−0.76, 0.04)	0.062
2pt static dom index	6.522	0.038^*^	0.408	−0.27 (−0.46, −0.08)	0.027	−0.22 (−0.57, 0.12)	0.176
2pt static Dom Ring	7	0.030^*^	0.438	−0.29 (−0.48, −0.10)	0.017^**^	−0.38 (−0.70, −0.05)	0.043
2pt Static non-dom index	2.273	0.321	0.142	−0.11 (−0.30, 0.08)	0.276	−0.10 (−0.78, 0.58)	0.395
2pt Static non-dom ring	2.24	0.326	0.140	−0.05 (−0.32, 0.23)	0.932	−0.24 (−0.63, 0.15)	0.172
BCB left index	0.929	0.629	0.058	0.10 (−0.48, 0.27)	0.345	0.04 (−0.46, 0.54)	0.833
BCB left ring	2.385	0.304	0.149	0.02 (−0.34, 0.38)	0.865	0.53 (−0.37, 1.43)	0.367
BCB right index	1.5	0.472	0.094	0.49 (0.02, 0.96)	0.035	0.48 (−0.30, 1.25)	0.176
BCB right ring	6	0.050^*^	0.375	0.17 (−0.16, 0.50)	0.439	0.54 (0.17, 0.90)	0.017^**^
Sentence jerk	4.571	0.102	0.327	−0.14 (−0.43, 0.16)	0.263	−0.28 (−0.62, 0.07)	0.091
Sentence pressure	0.857	0.651	0.061	−0.15 (−0.46, 0.16)	0.575	−0.19 (−0.64, 0.27)	0.310
Sentence velocity	3.429	0.18	0.245	6.68 (−5.34, 18.71)	0.208	−0.14 (−5.90, 5.62)	0.735
CSP	4.75	0.093	0.297	−0.06 (−0.25, 0.12)	0.441	−0.19 (−0.39, 0.01)	0.036
Emotional well-being	11.214	0.004^*^	0.701	0.06 (−0.06, 0.19)	0.161	0.17 (0.06, 0.28)	0.018^**^
Physical functioning	4.625	0.099	0.289	0.02 (−0.10, 0.15)	0.893	0.10 (−0.10, 0.29)	0.109
Role of limitations due to physical health	3.8	0.15	0.239	0.41 (−0.35, 1.16)	0.102	0.33 (−0.57, 1.24)	0.655
ADDS	6.091	0.048^*^	0.381	0.13 (0.00, 0.25)	0.028	0.18 (−0.11, 0.48)	0.116
WCRS movement score	5.636	0.06	0.352	3.33 (−0.61, 7.27)	0.157	1.25 (−0.88, 3.38)	0.109
WCRS writing speed	5.2	0.074	0.325	0.22 (−0.12, 0.56)	0.046	0.25 (−0.14, 0.64)	0.18

*CSP, cortical silent period; Dom, dominant; Non-dom, non-dominant; 2pt, two-point discrimination; ADDS, arm dystonia disability scale; BCB, Byl–Cheney–Boczai sensory discriminator; WCRS, writer's cramp rating scale. No significant interactions detected*.

* p < 0.05, and

** *p < 0.025*.

### Single subject analysis

**Figure 4:**
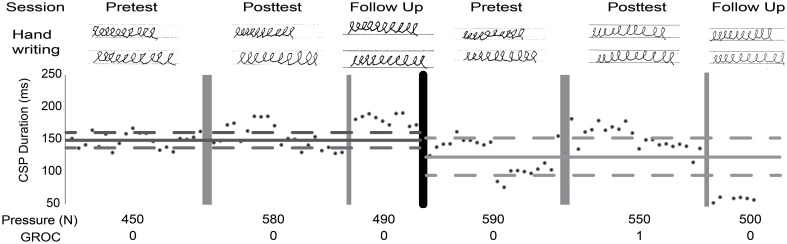
**Single subject analysis**. Raw data presented across both phases. Lines represent mean (solid) and two SD (dashed) of baseline; dark gray, SMR; light gray, CTL. Cortical silent period (CSP) duration, average handwriting pressure while drawing loops, Global rating of change (GROC) scores (0, change; 1, almost the same). Improved handwriting quality after both interventions was observed while pressure remained stable. CSP suggests a decrease in excitability at both post-tests and at SMR follow-up.

Given the small number of patients in this study and known variable response to rTMS, a single subject analysis was also done. The benefit of single subject analysis in small *n* clinical studies, is that it allows for detailed analysis of within subject variability and response that is masked by group level statistics (Kimberley and Di Fabio, 2010). All subjects displayed changes in at least one measure but changes did not consistently reflect improvement in all measures with rTMS + SMR training, as hypothesized (Table 5). Two subjects in the SMR group experienced a clinically meaningful improvement in function. One subject had evidence of handwriting improvement despite only reporting a minimal change in symptoms by the GROC and no change in handwriting pressure (Figure 4).

**Table 5:** **Single subject analysis for the first phase at post-test**.

**Subject**	**GROC**	**Physical function**	**CSP**	**WCRS**	**Sensory**	**Pressure**
1	+	−	−	0	0	+
2	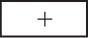	+	0	0	+	0
3	0	0	+	+	0	0
4	+	0	+	0	−	0
5	+	0	−	+	+	0
6	+	0	+	0	0	+
7	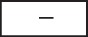	0	0	−	+	+
8	0	−	−	0	+	^*^
9	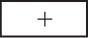	0	0	0	+	+

*Results are depicted with a (+) for an improvement in a testing component and a (−) for a worsening or change in the undesired direction. Clinically meaningful changes are outlined. Global rating of change (GROC), cortical silent period (CSP), writing movement score (WCRS), Two point discrimination static test on index affected hand (sensory), handwriting pressure during sentence writing (pressure)*.

* *denotes missing data as DSS08 did not complete digitized handwriting*.

## Discussion

The results suggest rTMS paired with sensorimotor training is not superior to rTMS with control. The findings further suggest a potential longitudinal benefit associated with two bouts of rTMS separated by 1 month, as indicated by subjective rating improvements.

The inhibitory rTMS applied here was intended to promote a favorable inhibited cortical state with rehabilitation applied during the improved neural environment following the neuromodulation. SMR was selected as a likely beneficial treatment because evidence of clinical improvement has been observed with this intervention (Byl et al., 2009). Yet, this therapy is very time intensive and may be challenging for patients to undertake, thus adjunct therapies that may enhance the effects of SMR would be advantageous. The SMR treatment performed here was brief in comparison to previous work, however which could have contributed to the lack of superior beneficial effect observed. Interestingly, despite the short duration of training, there was a significant improvement in dynamic sensory discrimination as measured by the BCB and two point static and dynamic discrimination testing on some fingers. This suggests that even a small amount of training may be able to improve this ability and it may be associated with perceived symptom improvement.

The control intervention was selected to deliver equal dose of intervention time and match investigator attention provided to the participant, but was not intended to be a specific therapy for FHD, as there is no evidence that general stretching and massage should improve FHD. Active digit and hand movement and strengthening were avoided as a control intervention, as it has been shown that there was no difference in improvement of FHD with either a task specific training or a more generic motor intervention (Zeuner et al., 2008). The evidence here is unable to determine if the control therapy gave additional benefit beyond what would be delivered with rTMS alone, as there was no group that received only rTMS.

It is interesting to note that there was no effect of increased inhibition after rTMS as measured by an increased CSP in the group analysis. Our single subject analysis suggests variable responses between subjects. It is important to note, however, that our measurements were taken at least 24 h after conclusion of the rTMS. It has been previously reported, that the increased inhibitory effect of rTMS may only be transiently observable and not persist beyond immediately post intervention (Kimberley et al., 2015). It is unknown if a more comprehensive TMS assessment may have revealed other changes.

Another consideration is that the training practice began immediately following the neuromodulation which may have influenced the potency of training effects, as there is evidence to support that a preceding bout of neuromodulation may alter the effect of motor training (Jung and Ziemann, 2009). Further, it is unknown what the ideal time frame is for rTMS effects to optimally manifest or whether treatment tasks should precede or follow the neuromodulation for the most effective capitalization of neuroplasticity. It is noteworthy to consider that the vast majority of literature characterizing the effects of non-invasive brain stimulation are based on a healthy response, yet it has been shown that people with dystonia have an abnormal plastic response to both behavioral and neuromodulatory perturbations (Weise et al., 2011; Meunier et al., 2012; Belvisi et al., 2013). This deficient homeostatic plasticity may then augment the cortical response secondary to rTMS combined with behavior training, unlike in a healthy individual. Future work should recognize that the ideal protocol for learning in a healthy individual may not clearly translate to an intervention in dystonia. Inclusion of a healthy control group may be advantageous to compare responses to neuromodulation interventions and further our understanding of how rTMS in healthy adults may support or differ in regards to persons with dystonia.

## Future work and limitations

The paramount limitation to this study was a small number of participants. The small sample may have underpowered the study and as a result, real change between groups went undetected. The high variance seen in our single subject analysis is not unusual as a current conundrum in neuromodulation is the variable response between subjects to many different types of neuromodulation (López-Alonso et al., 2014). Notably, individual participants did display significant changes in TMS outcomes as determined by the single subject analysis, yet when averaged as a group, the effects were nullified. Of note, however, is that the physiologic TMS measures did not agree with the clinical measures. This has finding has been supported in other work as well (Pomeroy et al., 2007; Kimberley et al., 2013; Sadnicka et al., 2014).

Additionally, self-rated improvements did not agree with other measures. This may indicate that the outcome measures used are not sensitive enough to capture the therapeutic effects; however, a placebo effect must also be considered. The likelihood of placebo effect is weakened though when one considers that the beneficial effect had a moderate effect size. A disagreement between objective measurement and self-rated improvement has been reported in musician's dystonia where patients tended to rate their improvement higher than an objective keyboard assessment task (Van Vugt et al., 2014). Van Vugt et al. (2014) speculated that this was due to patient self-selection of tasks during their day that less severely triggered dystonic symptoms, leading to an overestimation of ability. A similar phenomenon may be at play in these results.

Clinical studies in dystonia are often characterized by small samples and a heterogeneity of responses require careful consideration in the study design (Galpern et al., 2011). It is interesting that self-reported change continued to improve despite ongoing intervention. This finding suggests that the choice of a cross-over design in FHD or rTMS trials may not be ideal. This design was selected to maximize recruitment in a rare disease, reduce between subject variability, and give all participants the chance to experience the active intervention. Due to variability in the disorder on a day-to-day basis and variability in baseline TMS responses, a two-group design would better control for a potential carryover effect. Accordingly, factors that predict a participants' response should be further evaluated to investigate the role of non-invasive brain stimulation in those with focal dystonia and multicenter trials should be considered to achieve higher recruitment.

This design did not assess for potential benefit from the SMR alone with sham rTMS, nor a sham-sham effect (sham rTMS + control intervention). Given the current state of the literature that reports highly variable response to SMR training, and only after extensive practice, we felt it was unlikely that 30 min of training alone would be sufficient to demonstrate benefit, however this could be additionally investigated. Also, the effect of rTMS to PMC vs. sham rTMS has been studied with the same parameters as used in this trial, thus, if large physiologic or behavioral changes were observed a historical comparison could have been made (Murase et al., 2005; Borich et al., 2009; Kimberley et al., 2013, 2015). Finally, there is sufficient literature that suggests that in some people with FHD, inhibitory rTMS to the PMC may have beneficial effects, but other potential targets to stimulation should be explored.

## Conclusion

The results suggest rTMS paired with sensorimotor training is not superior to rTMS paired with control therapy, but there are potentially beneficial therapeutic effects of rTMS combined with non-specific rehabilitation in the treatment of FHD according to self-perceived measures. The majority of participants (6 of 9, 67%) reported improvement in self-rated function; however, two subjects experienced no change and one subjects reported worsening of symptoms. The variability of responses suggests rTMS interventions may not be effective in all participants with symptoms of dystonia and the optimal intervention and timing of synergist intervention remains to be determined.

### Conflict of interest statement

The authors declare that the research was conducted in the absence of any commercial or financial relationships that could be construed as a potential conflict of interest.
